# Implementation of a patient reminder system in Haiti in a socio-political crisis context: evaluation of outcomes

**DOI:** 10.1186/s12913-024-11395-0

**Published:** 2024-08-20

**Authors:** Marcmy Presume, Charles Patrick Almazor, Mathias Altmann

**Affiliations:** 1 Association de Technologistes Médicaux Licenciés d’Haiti (ATMLH), Port-au-Prince, Haiti; 2 Hopital Sage Citymed, Port-au-Prince, Haiti; 3 University of Bordeaux, National Institute for Health and Medical Research (INSERM) UMR 1219, Research Institute for Sustainable Development (IRD) EMR 271, Bordeaux Population Health Research Centre, Bordeaux, France

**Keywords:** Program evaluation, Patient appointment, Reminder systems, Text messaging

## Abstract

**Background:**

In Haiti, patient’s adherence to treatment and compliance with medical appointments are very challenging due to different local factors. We aimed to assess the effectiveness of a reminder system implemented in health facilities in Haiti in a context of socio-political crisis.

**Methods:**

*We used* appointment data *from patients aged 15 years and older between January 2021 and November 2023 from four healthcare centers in the Port-au-Prince metropolitan area. We performed descriptive analysis*,* crossing covariates with appointment attendance. We performed Pearson’s Chi-squared test*,* and multivariate regression analysis using a mixed-effect logistic regression model in order to explore the association between sending reminders and appointment attendance*,* with and without adjustment for other patient-level covariates.*

**Results:**

A total of 14 108 appointments were registered on the reminder systems, with 2 479 (17.6%) attendances. Among those to whom reminders were sent, the number of attendances was 167 (17.4%) for email recipients only, 199 (36.7%) for SMS recipients only, and 19 (42.2%) for both SMS and email recipients – versus 2 094 (16.7%) for non-reminders. After adjusting on all other covariates, we found that patients to whom a reminder was sent via email (aOR: 1.45; CI: 1.08, 1.94), SMS (aOR: 2.95; CI: 2.41, 3.60), and both SMS and email (aOR: 2.86, CI: 1.37, 5.96) were more likely to show up on their appointment day compared to those who did not receive any reminder. Other socio-demographic factors such as being 50 years and older (aOR: 1.31; CI: 1.10, 1.56) compared to under 30 years, living as couple (aOR: 1.23; CI: 1.10, 1.37), and not having children (aOR: 1.21; CI: 1.07, 1.37) were significantly associated with appointment attendance.

**Conclusions:**

Our study suggests that patient reminder systems may be used to reduce non-attendance in Haiti, even in a context of socio-political crisis.

## Background

Non-attendance is a major public health challenge for healthcare systems in both high- and low-income countries. It is very costing for healthcare providers as well as for patients, creating inefficiencies, keeping inactive the utilization of valuable resources, and disrupting the healthcare delivery system [1–5]. Non-attendance may favor non-adherence to treatment among patients, increase dissatisfaction, reduce their quality of life, and may affect other patients’ health who may need an earlier appointment [5]. 

Reminder systems have been used around the world to face non-attendance challenges. There are various types of reminder systems based on the channel used such as telephone call, voice mail, short message service (SMS), email, postal letter, simple immediate notification at the time of scheduling the appointment, and they could be manual or automated [6–9]. However, their effectiveness mostly depends on the use-case and the context such as the patient population, the modality of reminder, and the service type [10]. 

Effectiveness of text messaging reminder systems have been proven in various domains such as postnatal care, infectious and chronic disease treatment, and for different purposes including improving adherence to treatment and reducing non-attendance [6, 11–13]. But most of those studies have been conducted in high income countries. Liu et al., in their meta-analysis on reminder systems to improve patient adherence to tuberculosis clinic appointments for diagnosis and treatment, recommend future studies of modern technologies such as SMS reminders, particularly in low-resource settings [12]. To our knowledge, such systems were not previously evaluated in a socio-political crisis context.

Haiti is known for its protracted crisis, with political instability and frequent natural disasters. Only one presidential election occurred in the country for the past 10 years, and the elected president was murdered in July 2021, the last year of his mandate. Since then, the crisis worsened, making daily life very complicated. A lot of people were displaced, and thousands of Haitians fled from the country [14–16]. Gang violences have also limited access to healthcare services [14, 17]. Due to the socio-political crisis, terrorism attacks, repeated violences on healthcare professionals, and different attacks on health facilities, patient’s adherence to treatment and compliance with medical appointments are very challenging [18, 19]. 

Mobile phones are very popular in Haiti. The International Telecommunication Union reports that 58.7% of the population in the country owned a mobile cellular telephone in 2018 while the proportion of the population aged 18 years and older was 57.7% according to the last estimation of the population in 2015 by the Haitian Institute of Statistics and Informatics (IHSI) [20, 21]. Communication technologies like text messaging as part of a reminder system could help improve patient’s care in the country.

Six years ago, some healthcare centers in Haiti started using an integrated patient reminder system whose objective is to reduce non-attendance by increasing the number of patients showing up on their appointment day. Through this study, we aimed to assess the effectiveness of this reminder system to figure out whether sending reminders improved attendance in the context of a socio-political crisis.

## Methods

### Study setting

**Fig. 1:**
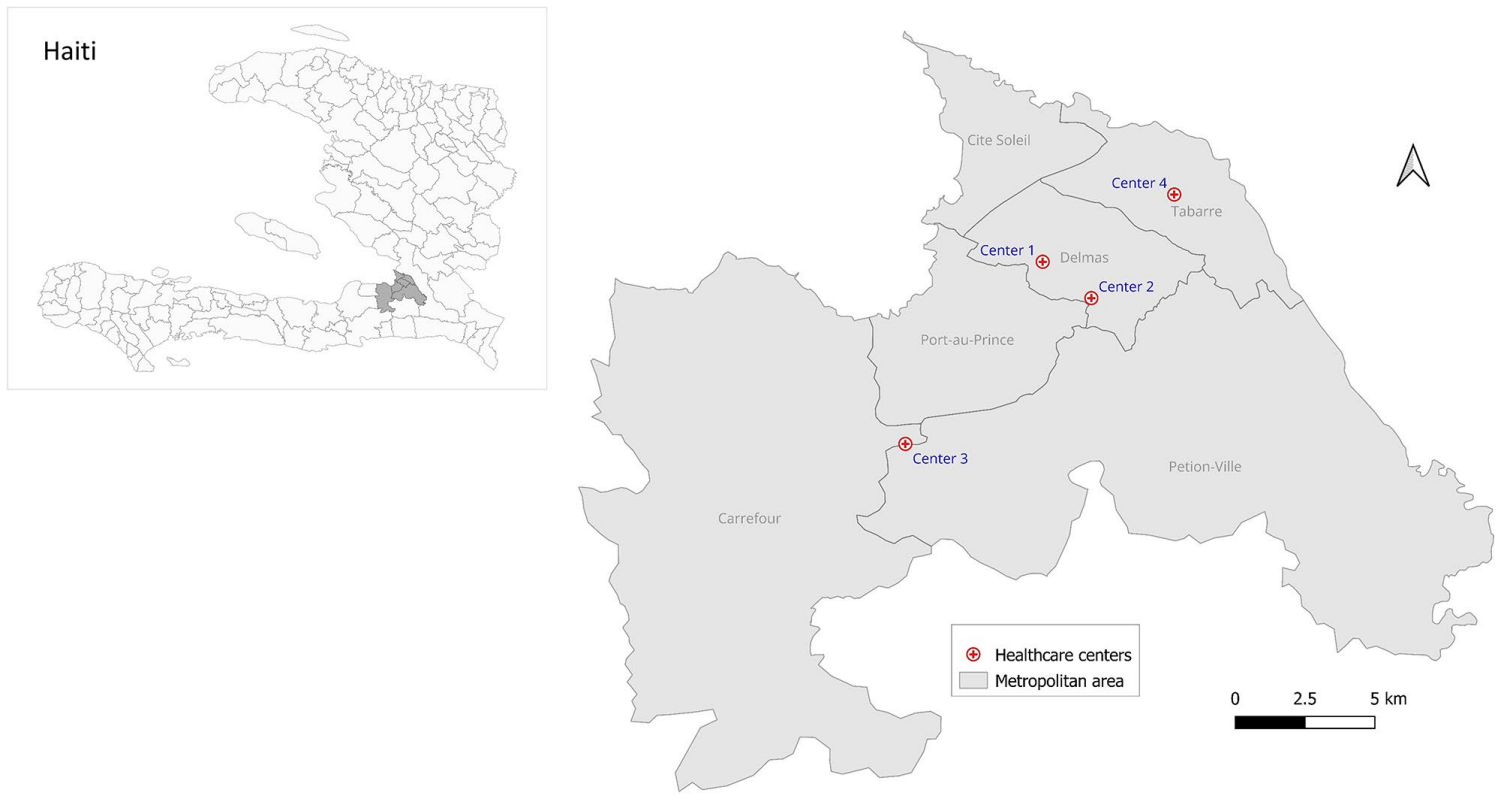
Geographic location of the four healthcare centers included in the study, Haiti, 2023

In order to assess the effectiveness of the reminder system, we selected the seven healthcare centers in the Port-au-Prince metropolitan area (known as metropolitan area) who implemented the system, but only four of them volunteered to participate. We limited the study in the metropolitan area because about 80% of killings and injuries were registered in this area, and we wanted a homogenous sociopolitical environment and access to care (in termes of distance) [14]. Among the four healthcare centers that volunteered to participate, two are located in Delmas, one in Petion-ville, and one in Tabarre (Fig. 1). The first included healthcare center is a 37-bed facility, with a package of services including internal medicine, surgery, orthopedics, pediatrics, gynecology, emergency care, laboratory tests, pharmacy, and imagery. The center is located in Delmas and first implemented the system in January 2021. The second one is a 24-bed facility with a package of services including internal medicine, surgery, orthopedics, pediatrics, gynecology, urology, otorhinolaryngology, emergency care, laboratory tests, pharmacy, and imagery. The center is located in Delmas and first implemented the system in April 2022 and used it until the beginning of 2023. The third one is a 20-bed facility, with a package of services including internal medicine, surgery, odontology, ophthalmology, pediatrics, maternity (gynecology and prenatal care), emergency care, laboratory tests, pharmacy, and imagery. The center is located in Petion-Ville and fist implemented the system in August 2023. And the fourth one is an ambulatory clinic, with a package of services including internal medicine, pediatrics, gynecology, laboratory tests, and pharmacy. The center is located in Tabarre and first implemented the system in May 2021. Except for the one in Tabarre, which only accepts outpatients, the other three healthcare centers accept both inpatients and outpatients.

### The reminder system

In Haiti, the reminder system has been implemented since October 2017 in 20 private healthcare centers using a healthcare management system. Among those 20 healthcare centers, seven are in the metropolitan area. The healthcare management system is an all-in-one system including an electronic medical record, a laboratory information system, and other modules adapted to healthcare systems such as accounting, stock management, personal management, etc. The healthcare management system includes an appointment module as well. Patients first arrive to the healthcare centers by drop in visit, and the healthcare centers use the reminder system for the next visits. Reminders are sent by E-mails 24 hours before appointment dates to patients who provided an electronic email address. The system may send SMS reminders to patients as well. When sending SMS, users (medical staff) do not choose recipients. The system administrator at each healthcare center must add funding to its SMS account on a regular basis to support automated SMS messaging. The system then sends the reminders to all scheduled appointments on their scheduled and recall date until the healthcare center’s SMS account is empty.

### Study design

Our observational study used existing data from a routinely-used appointment reminder system to assess the effectiveness of the reminder system in improving appointment attendance. We used appointment data from patients aged 15 years and older registered between January 2021 and November 2023 from four healthcare centers in the metropolitan area. The reminder system sends automatic reminders via email or SMS to patients before their appointment day. Email reminders were free of charge for both healthcare centers and patients, but not all patients had an email. SMS reminders were free of charge for patients but had a cost for healthcare centers and the system administrators needed to recharge the SMS account of the system in order for SMS to be sent. All patients had a phone number, but SMS were not sent to all of them since the healthcare centers did not always recharge their SMS account on time. The primary outcome was the attendance at the scheduled appointment. We compared the attendance rate among those to whom reminders were sent before the appointment day and among those to whom no reminder was sent to figure out whether sending reminders improved attendance. The analysis was only among those were eligible to receive reminders based on having a cell phone, having an email, or both. Within our overall inquiry about the effectiveness of the reminder system, we explored temporal trends in appointment attendance as compared to temporal trends in fatalities from civilian targeting events.

### Population and covariates

In this study, we included appointments from patients aged 15 years and older registered on the reminder system in the included healthcare centers from January 2021 to November 2023. Those appointments were recorded by medical staff, mostly physicians, after each consultation session. We extracted patients’ socio-demographic characteristics, reminder information, and attendance status from these appointments. The dependent variable was appointment attendance (patient showing up on their appointment day). The covariates included means of reminder (email, SMS, both SMS and email, or no reminder); gender (male or female); age (being under 30 years old, 30 to 40 years, 40 to 50 years, or 50 years and older); living in the metropolitan area; living as couple (we considered living as couple, if the patient is married or living in cohabitation with his/her partner, otherwise: no); and having children (having at least one child).

In addition to appointment data, the number of fatalities from civilian targeting events in the metropolitan area was collected from Armed Conflict Location & Event Data Project (ACLED). ACLED used traditional media, reports from International institutions and non-governmental organizations, data from local partner, and social media (targeted and verified) as sources of information [22]. 

### Statistical analysis

We described the reminder system usage. We performed temporal description of appointments, attendances, reminders, and fatalities from civilian targeting events. Smooth curves were drawn using locally estimated scatterplot smoothing (loess) method. We described appointment attendance according to the other covariates. We performed Pearson’s Chi-squared test in order to figure out whether there were relationships between covariates and appointment attendance. In order to figure out whether sending reminders and other covariates had independently an impact on appointment attendance, we performed a multivariate regression analysis using a mixed-effect logistic regression model. We included patients and healthcare centers as random effect, assuming that a patient could have more than one appointment during the study period, and patients’ decision to attend their appointments may be influenced by the healthcare center.

We used R version 4.2.2 with the lme4, gtsummary, and ggplot2 packages for statistical analysis.

## Results

**Fig. 2:**
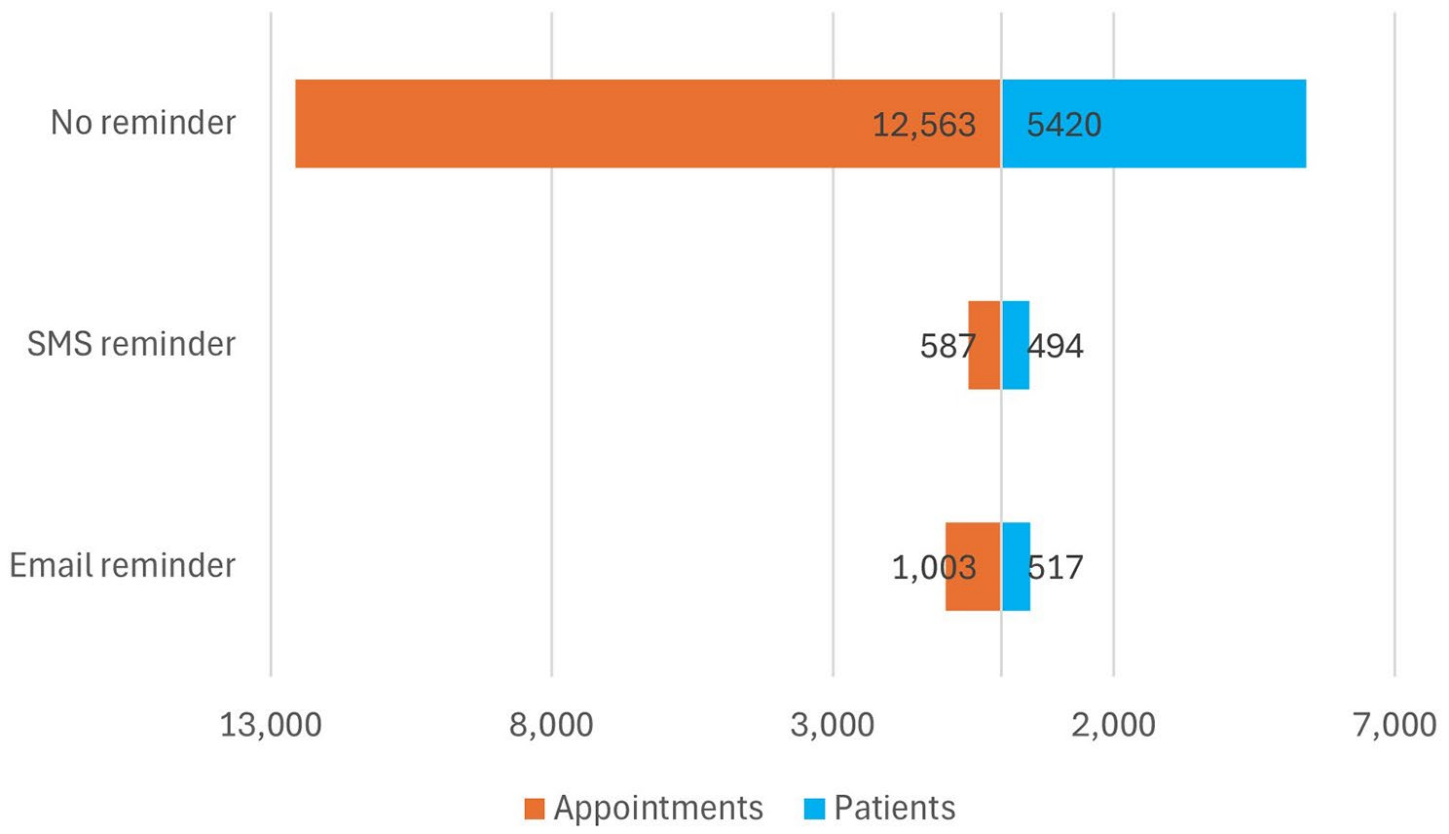
Reminder system usage, Haiti, from 2021 to 2023

Between January 2021 and November 2023, 14 108 appointments from 6 401 distinct patients aged 15 years and older were registered on the reminder systems of the four included healthcare centers, with an average of 2.2 appointments per patient. A total of 1003 (7.1%) reminders were sent via email to appointments and the number of unique patients related to those appointments was 517 (8.1%). Only 587 (4.2%) reminders were sent via SMS to appointments and the number of unique patients was 494 (7.7%) (Fig. 2).

*In orange*,* on the left: total number of appointments by reminder means. In blue*,* on the right: total number of unique patients by reminder means.*

**Fig. 3:**
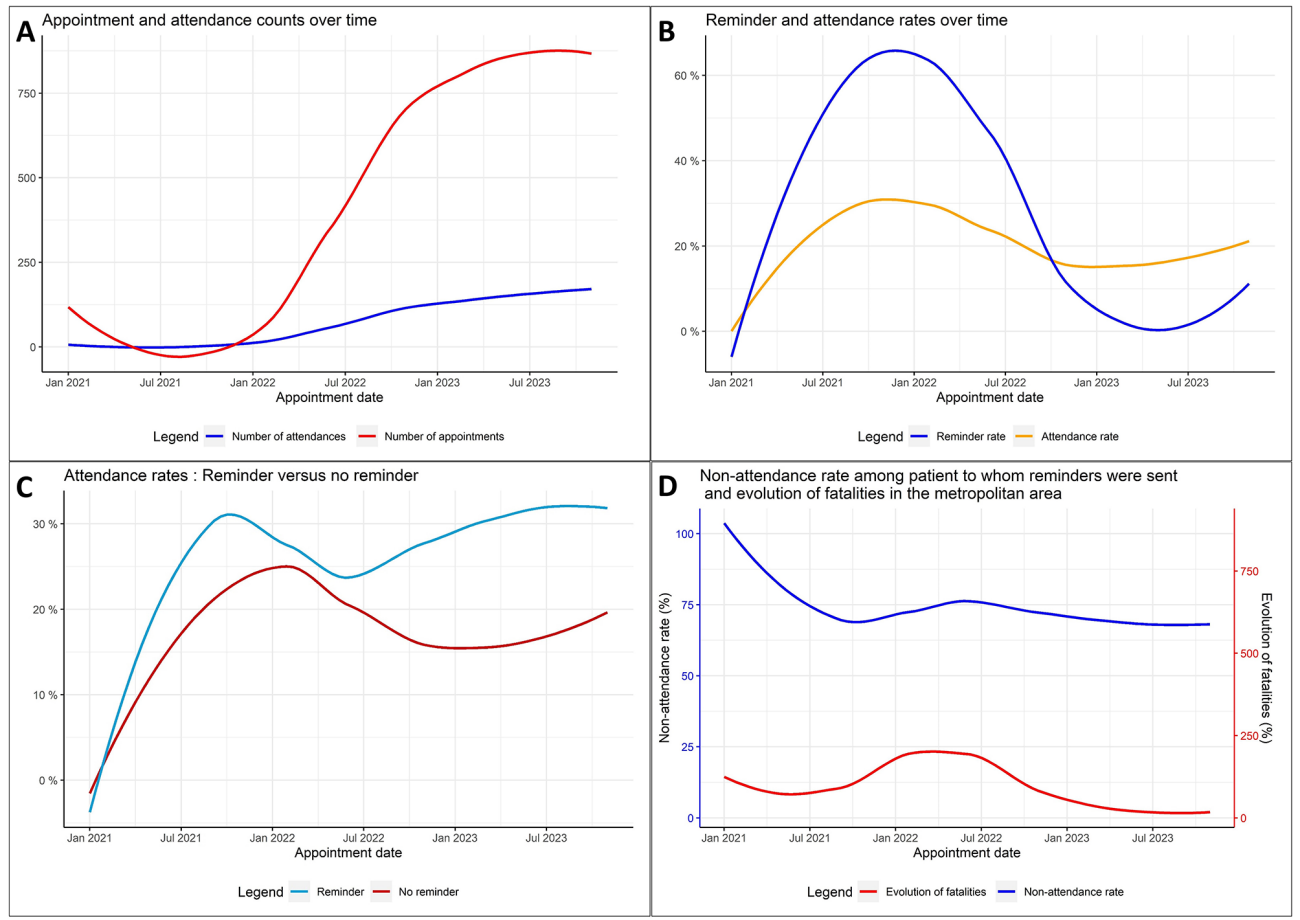
Attendance and fatality from civilian targeting events, Haiti, from 2021 to 2023. **A**: Attendance and appointment curves. In blue, the number of attendances curve; in red, the number of appointments curve. **B**: Reminder and attendance rates curves. In blue, reminder rate curve; in orange, global attendance rate curve. **C**: Comparison between attendance rates curves for patients to whom reminders were sent and those to whom no reminder was sent. In green, attendance rate curve for patients to whom reminders were sent; in dark red, attendance rate curve for patients to who no reminder was sent. **D**: Non-attendance rate among patients to whom reminders were sent and evolution of fatalities from civilian targeting events in the metropolitan area compared to the previous month. In blue, curve of non-attendance rate among patients to whom reminders were sent linked with percentage values on the primary axis; in red, curve of the evolution of fatalities compared to the previous month linked with percentage values on the secondary axis

The attendance and appointment curves followed the same trend, with very low appointments in 2021 followed by an exponential growth of appointments and attendances from 2022. The rate of reminders sent followed the same trend as the attendance rate over the study period. The rate of reminders sent was higher than the attendance rate before September 2022, and dropped lower after this date. Splitting the attendance rate according to the reminder status showed a higher attendance rate over time for patients to whom reminders were sent compared to patients to whom no reminder was sent. The non-attendance rate among those to whom reminders were sent followed slightly the same trend as the evolution of fatalities from civilian targeting events (Fig. 3).

Of the 14 108 appointments, 83.5% were for female patients, 40.5% were for patients aged 30 to 40 years, 98.9% were for patients living in the metropolitan area, 51.6% were for patients living as couple, and 55.2% were for patients who had no children (Table 1).

**Table 1:** Patient demographic characteristics associated with appointments from January 2021 - November 2023 (*n* = 14,108)

	Sample size *N* = 14 108	%
Gender		
Male	2 327	16.5
Female	11 774	83.5
Age		
Under 30 years	4 210	30
30 to 40 years	5 685	40.5
40 to 50 years	1 973	14
50 years and older	2 184	15.5
Living in the metropolitan area		
No	153	1.1
Yes	13 955	98.9
Living as couple		
No	6 773	48.4
Yes	7 208	51.6
Having Children		
No	7 780	55.2
Yes	6 322	44.8

The total number of attendances observed was 2 479 (17.6%). Of the reminders sent, 958 reminders were sent via email only and the number of attendances among them was 167 (17.4%), 542 were sent via SMS only and the number of attendances among them was 199 (36.7%), and 45 were sent via both email and SMS and the number of attendances among them was 19 (42.2%). A total of 12 563 patients did not receive any reminder before the appointment day because they did not have an email address and the healthcare center did not have credit to send SMS reminders to them during their appointment period. The number of attendances among them was 2 094 (16.7%). Attendance rates were statistically significantly different by reminder status (no reminder vs. email reminder vs. SMS reminder vs. both email and SMS reminder) (*p*-value < 0.001). Regarding patients’ socio-demographic characteristics, the number of attendances was 769 (18.3%) among under 30 years versus 1 002 (17.6%), 279 (14.1%), 419 (19.2%) among 30 to 40 years, 40 to 50 years, and 50 years and older, respectively (*p*-value < 0.001). Among those who are living alone, the number of attendances was 1 121 (16.6%) versus 1 330 (18.5%) among those who are living as couple (*p*-value = 0.003). Among patients who have children, the number of attendances was 1 045 (16.5%) versus 1 433 (18.4%) among those who do not have any child (*p*-value = 0.003) (Table 2).

**Table 2:** Patient characteristics and attendance rates

	Number of attendances (*n*)	Number of appointments (*N*)	Attendance rate (%)	*p*-value^1^
Sample	2 479	14 108	17.6	
Reminder means				< 0.001
No reminder	2 094	12 563	16.7	
Email	167	958	17.4	
SMS	199	542	36.7	
SMS and email	19	45	42.2	
Gender				0.038
Male	374	2 327	16.1	
Female	2 103	11 774	17.9	
Age				< 0.001
Under 30 years	769	4 210	18.3	
30 to 40 years	1 002	5 685	17.6	
40 to 50 years	279	1 973	14.1	
50 years and older	419	2 184	19.2	
Living as couple				0.003
No	1 121	6 773	16.6	
Yes	1 330	7 208	18.5	
Having Children				0.003
No	1 433	7 780	18.4	
Yes	1 045	6 322	16.5	

^1^Pearson’s Chi-squared test

**Fig. 4:**
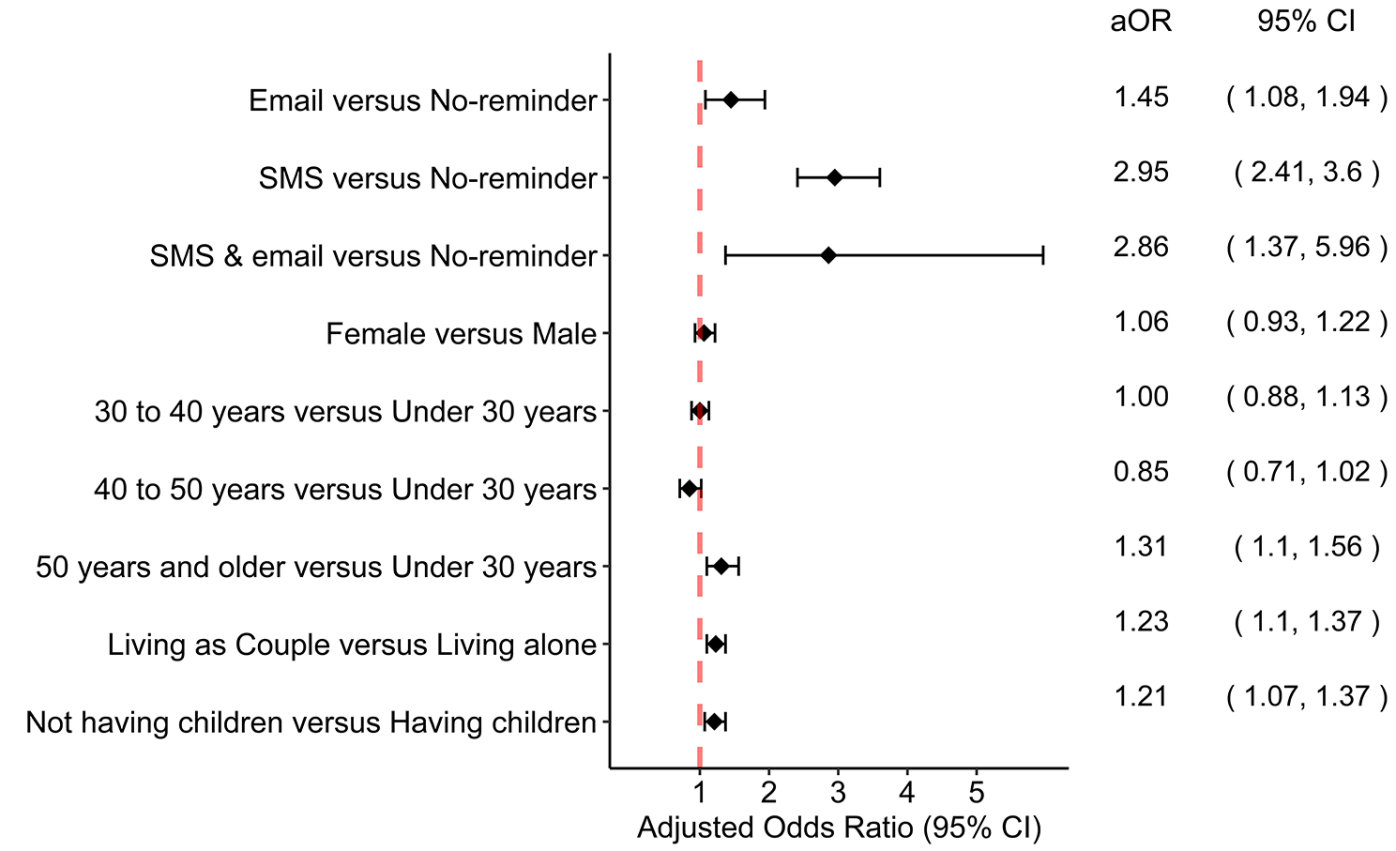
Factors associated with appointment attendance, Haiti, from 2021 to 2023. aOR: adjusted Odds Ratio. CI: Confidence Interval

In the multivariate analysis, we found that patients to whom a reminder was sent via email (aOR: 1.45; CI: 1.08, 1.94), SMS (aOR: 2.95; CI: 2.41, 3.60), and both SMS and email (aOR: 2.86, CI: 1.37, 5.96) were more likely to show up on their appointment day compared to those who did not receive any reminder. Other socio-demographic factors such as being 50 years and older (aOR: 1.31; CI: 1.10, 1.56) compared to under 30 years, living as couple (aOR: 1.23; CI: 1.10, 1.37), and not having children (aOR: 1.21; CI: 1.07, 1.37) were significantly associated with appointment attendance. Female gender (aOR: 1.06; CI: 0.93, 1.22) was not associated with appointment attendance (Fig. 4).

## Discussion

This multicenter study on the effectiveness of a patient reminder system to reduce non-attendance rate in the complex socio-political crisis of Haiti showed that patients receiving a reminder before their appointment day were more likely to attend their appointment compared to those who did not receive any reminder. The comparison of the attendance rate curves among patients to whom reminders were sent and those to whom no reminder was sent showed a difference that remained over time. The SMS group had an odds of attendance that was more than twice as high as the comparison group who received no reminders. Our results are in agreement with a meta-analysis assessing the effects of mobile phone messaging reminders for attendance at healthcare appointments that included eight randomized controlled trials from both high and low income countries with 6 615 participants [23]. Authors reported that mobile text message reminders significantly improved attendance rate at healthcare appointments compared to no-reminders (RR: 1.14; CI: 1.03, 1.26).

Appointment attendance depends on socio-demographic factors as well, independently of receiving reminders or not. Patients aged 50 years and older were more likely to show up on their appointment day. This result may be explained by the fact that older people have weaker health conditions and are more in need of health care than younger people. Other reasons explaining this result might be the fact that older patients are more conscientious about their health conditions and more afraid about dying. A study on follow-up non-attendance after long-bone fractures in a semi-urban city in Nigeria has found that patients were significantly younger in the non-attendance group than the attendance group (OR: 0.98; CI: 0.963, 0.996) [24]. Patients who are living as couple were more likely to show up on their appointment day. The presence of the other partner in a household appears to be very supportive for the patient. Studies conducted in Nigeria and in other countries have found significantly higher attendance rates among married patients than unmarried patients [24–26]. Patients who do not have children were more likely to show up on their appointment day. This is probably because they are less busy and have less socio-economic responsibilities. A cross-sectional study aiming to identify risk factors and patient-reported reasons for nonattendance at outpatient clinic appointments in human immunodeficiency virus-infected population has found that having children under 12 years old was a risk factor for non-attendance at medical appointment (OR: 2.11; CI: 1.10, 4.06) [27]. 

We found very low attendance rates in our study. The highest attendance rate was 42.2% and was for the group receiving both SMS and email. In the meta-analysis on the effects of mobile phone messaging reminders for attendance at healthcare appointments previously cited, attendance rates were between 67.8% and 80.6% [23]. This result may be partly explained by patient characteristics. Around 85% of patients included in the study were under 50 years. But their impact was too low to explain the low attendance observed. This low attendance is most probably due to the ongoing socio-political crisis in Haiti. The non-attendance rate among patients to whom reminders were sent followed the same trend as the evolution of fatalities in the metropolitan area. Because of high risk of kidnaping and frequency of terrorism events by armed gangs, and attacks on hospitals, people cannot freely move from one place to another, making access to vital facilities such as healthcare centers very challenging [18, 19, 28]. Socio-political crisis has a known impact on hospital attendance. It has been previously reported in Haiti in 2004 [29], and in other contexts like in Cameroun as well between 2018 and 2019 [30]. However, our study is based on the attendance on the scheduled appointment day only. A study on missed appointments among patients with stable chronic conditions in South Africa between 2014 and 2015 suggest that 67% of patients who missed original appointments presented voluntarily on a later date to obtain medicines [31]. 

We observed that the reminder system was gradually implemented in the corresponding healthcare center, mostly in 2021. It was effectively used starting from 2022. The usage rate of the reminder system was very low. Email and SMS reminders were sent to only 7.1% and 8.1% of the appointments, respectively. The low number of reminders sent by email can be explained by the low rate of patients having an email among the patients coming to the healthcare centers. The low number of SMS sent may be related to some other factors, such as the cost of SMS for the healthcare centers or the socio-political crisis, where recharging the system’s SMS account may have been challenging due to the situation. Despite the low usage rate, the reminder system has improved attendance overall.

Our study is the first to assess the effectiveness of a patient reminder system in a context of socio-political crisis, adjusting on socio-demographic covariates influencing appointment attendance. However, there were some limitations. SMS reminders relayed on the ability of the healthcare centers to recharge their SMS account. Healthcare centers that were less able to recharge their SMS accounts due to lack of funds or logistical challenges might also have a disproportionate share of disadvantaged patients who were less likely to attend appointments based on their inherent characteristics. However, since this factor is linked to the healthcare center itself, the resulting bias was reduced by adding the healthcare center as random effect in the multivariate regression analysis. We did not consider distance to healthcare centers, and cost of service packages because they were not available. Those parameters can influence appointment attendance and vary from one healthcare center to another and from one patient to another. However, the study was conducted in the metropolitan area, not in rural context, and the introduction of healthcare centers as random effects in the model reduced this bias.

## Conclusions

Our study showed that the patient reminder system might have contributed to reduce non-attendance rates in the Port-au-Prince metropolitan area, despite its suboptimal implementation. This suggests that patient reminder systems may be used to reduce non-attendance in the county, even in a context of socio-political crisis.

## Data Availability

The datasets used and/or analyzed during the current study are not publicly available due to privacy policy but are available from the corresponding author on reasonable request. However, prior authorization from related healthcare centers to access those data is mandatory.
